# Determinants for hesitancy in human papillomavirus (HPV) vaccine uptake among school girls in Jimma Town, Ethiopia. A mixed approach: quantitative and qualitative

**DOI:** 10.1186/s12978-023-01711-y

**Published:** 2023-12-01

**Authors:** Meron Dera, Abigiya Wondimagegnehu, Zeytu G. Asfaw

**Affiliations:** 1 Department of Epidemiology and Biostatistics, School of Public Health, Addis Ababa University, Addis Ababa, Ethiopia; 2 Institute of Medical Epidemiology, Biostatistics and Informatics, Martin-Luther-University, Halle, Germany

**Keywords:** HPV, Vaccine, Hesitancy

## Abstract

**Background:**

The human papillomavirus (HPV) vaccination helps the body fight off certain types of the virus. Despite being one of the top 10 health hazards in the world, vaccination hesitancy has received little attention in Ethiopia. In Jimma Town, Ethiopia, the current study aims to identify the variables that affect HPV vaccine resistance and evaluate HPV uptake resistance and related variables among female school students.

**Methods:**

A mixed study of quantitative and qualitative data collection and analysis were considered. An institution-based cross-sectional study was conducted from December 2022 to June 2023. Following a thorough random sampling process, 373 respondents were selected using stratified sampling techniques. The necessary information was gathered using an in-depth interview, a structured questionnaire administered using Kobotoolbox tools, and an interviewer who had undergone training. Models of multivariable and bivariate logistic regression were both used.

**Result:**

A total of 369 respondents participated in the study and the response rate was 98.9%. The hesitancy of the HPV vaccine in Jimma Town female school students was 39.02%. Female students who have a mobile phone, (OR = 0.46, 95% CI (1.16, 45.89), mothers educational status ($\ge$ Secondary) (OR = 0.53, 95% CI (1.11, 2.44), older sister vaccinated (OR = 0.19, 95% CI (0.004, 0.42), previously vaccinated two doses of HPV (OR=0.64, 95% CI (0.006, 0.137), Confidence (worried in safety and efficacy of vaccine) (OR = 3.42, 95% CI (0.30, 0.87), Not Belief in rumors (HPV vaccine ruins girls fertility) (OR = 0.21, 95% CI (0.43, 0.96), and Complacency (Distrust in health care information ) (OR = 0.24, 95% CI (0.37, 0.94), were found to be statistically significant with HPV Vaccine Hesitancy.

**Conclusion:**

Due to widespread misinformation in the community, both schoolgirls and parents had high levels of hesitancy for the HPV vaccine in this study compared to a similar study. As a result, the HPV vaccine program, with the help of the appropriate health care professionals, should work hard to maximize community awareness in order to significantly increase the uptake of the HPV vaccine.

## Introduction

Vaccines have protected more lives than any other scientific breakthrough. In 2006, the FDA authorized the first preventive Human papillomavirus (HPV) vaccination, Gardasil, which was sold by Merck and Co. According to a Merck press release, it had been approved in 80 countries by the second quarter of 2007, with many of them undergoing fast-track or expedited review. Several low-income African countries, notably Ethiopia, have started giving out the HPV vaccine with the assistance of the GAVI Alliance [1]. Belonging to the Papillomaviridae family, HPV is the most common sexually transmitted infection (STI). The group of viruses known as HPV has more than 100 different types of viruses. More than 40 different types of HPV can be spread through sexual contact. Most HPV-positive people don’t exhibit any symptoms. The HPV vaccine is available to females between the ages of 9 and 26. By the time they are 11 or 12 years old, girls should have received three doses of the HPV vaccine. If they did not receive any, all, or any combination of the doses when they were younger, they can receive the vaccine from the ages of 13–26 [2]. In addition to cancers of the vulva, vagina, penis, and anus, HPV can also lead to cervical cancer. Oropharyngeal carcinoma, a type of throat cancer, has also been linked to it. This includes the base of the tongue and the tonsils. The HPV vaccine is safe and effective in preventing HPV-related illnesses, such as cervical cancer, when given at the recommended ages [3]. Recurrence is a potential after cervical cancer has been identified and treated, and the impact of cervical cancer immunotherapy is constrained [4–6]. Of course, vaccine hesitancy is a severe problem despite the fact that treatment vaccines can teach the immune system to recognize and respond to these antigens and eliminate cancer cells that contain them. The present paper’s main goal is to comprehend the vaccine hesitation level and discover its causes.

HPV, which also significantly contributes to other anogenital and oropharyngeal cancers, is the primary cause of nearly all occurrences of cervical cancer. HPV causes 4.5 % of all cancers worldwide (630,000 new cases per year): 8.6 % of cancers in women and 0.8 % of cancers in men. Cervical malignancies brought on by HPV account for two-thirds of all cases worldwide [7–10]. Eight out of ten people will contract HPV at some point in their lives, and 80% of women will develop at least one kind of HPV throughout the course of their lifetimes [11].

In actuality, the HPV virus does not affect all regions of the world equally. Its distribution shows significant regional diversity. Sub-Saharan Africa (24%) and Latin America and the Caribbean (16%), Eastern Europe (14%) and South-East Asia (14%) had the highest rates of cervical HPV among women [12]. Though Ethiopia has a population of 36.9 million women ages 15 years and older who are at risk of developing cervical cancer but actual Data is not yet available on the HPV burden in the general population of Ethiopia. Besides, Ethiopia is one of the resource-limited countries, access to cervical cancer screening is very less (below 2% among cervical screening eligible women) and human papillomavirus infection is mostly asymptomatic and causes cervical cancer mainly after 20 years [13].

According to extremely limited information, cervical cancer is the second most common malignancy among Ethiopian women between the ages of 15 and 44. Pertaining to current estimates, every year 7445 women are diagnosed with the ailment, and 5338 of them pass away as a result of it [13–15]. A prior survey in Jimma Town found that just half of the respondents (52.7%) and almost one-third (31.4%) had enough understanding of and favorable perspectives on HPV vaccination, respectively [14] and the researchers say that other researchers focus on the reasons for why attitudes toward the vaccine decreasing. According to another study conducted in Jimma Town, 68.9% of survey respondents who said they would be receptive to receiving HPV vaccines suggested that only two-thirds of survey respondents were eager to get inoculated. Although the HPV vaccination is freely available, researchers strongly urged undertaking reasonable number of researchs on the reasons why people don’t want to accept it, as this would increase the amount of vaccine reluctance [14].

Besides, more than a quarter of the 400 parents in Addis Abeba who took part stated they had no knowledge of the HPV vaccine, and more than a third indicated they disapproved of it [14]. Based on the aforementioned facts, there is misunderstanding about HPV vaccination in both school girls and their parents where the present paper need to target these population. Of course, the federal government of Ethiopia has paid attention but the COVID-19 epidemic has made immunization efforts much more challenging. Ethiopia started a vaccination campaign in 2021 for girls who missed one or both doses due to school closings. Ethiopia advises HPV immunization for females aged 9 to 14 years before they start having sexual relations [15]. According to a variety of academic sources, there are three main obstacles preventing women from getting immunizations. First, gender-based barriers including false beliefs about how vaccinations impact women. Second, there are constraints based on societal conventions, and females are reluctant to go to health facilities. Third, there are logistical and educational difficulties as well, as many individuals are not aware of the advantages of the HPV vaccine or how to get it. Thus, the purpose of the present study is to provide light on the factors that led to the hesitation of female students and the factors that influenced their decision to get immunized.

Vaccine hesitancy is one of the top 10 risks to world health in 2019. According to the Strategic Advisory Group of Experts (SAGE) Working Group, Vaccine hesitation is described as a delay in acceptance or a refusal to accept vaccination despite the availability of the vaccine [16]. SAGE is tasked with advising WHO on global policies and strategies that cover a wide range of topics, including vaccines and technologies, research and development, immunization delivery, and its connections to other health treatments. Factors such as complacency, constraints, and confidence all have an impact on it. Immunization reluctance has a direct impact on vaccine uptake and coverage rates. Vaccine hesitancy is complex and context-specific, varying through time, space, and vaccines [17]. As part of excellent program practices, nations should implement a method for measuring and handling vaccine hesitation in their nationwide vaccination program. To the researcher’s knowledge in the research area, as well as Ethiopia, there are no studies on vaccine hesitancy and related factors with relation to the HPV vaccine. Hence, the present study aimed at putting a plug in this gap and reducing morbidity due to HPV and related diseases [18].

## Methods

### Study area and study period

The research was conducted at selected Jimma town primary and secondary schools from December 2022 to May 2023. Both public and private schools were considered. According to the 2007 census, it has a population of 120,960, of which 60,136 were women. Besides, it has 35 public primary and secondary schools and also, 37 private primary and secondary schools. Jimma is the largest city in southwestern Oromia and is located 352 kms to the southwest of Addis Ababa.

### Study design

A cross-sectional study that combined quantitative and qualitative methods was carried out. A qualitative inquiry was used in-depth interviews to explain reasons provided via free text responses to describe perspectives about the Human Papillomavirus, vaccination hesitancy, and its associated factors in Jimma town school girl parents. An institution-based analytical cross-sectional quantitative study design was used to investigate associations between reasons categories and socio demographic variables of special interest in Jimma town school girls.

### Study population and study unit

The sample populations: Selected Female students who were attending schools during the study period at Jimma town.

The study units: all female students who registered at the chosen institution.

### Inclusion and exclusion criteria

Inclusion criteria: During the time of data collection, all female students who attended the chosen school.

Exclusion criteria: This study excluded female schoolgirls who were eligible for the HPV vaccine but did not obtain parental or caregiver consent.

### Ethical considerations and procedures

During the study, basic ethical research concepts such as informed consent, confidentiality, beneficence, non-malfeasance, and justice were all taken into consideration. The ethical review committee of the AAU received this study for approval. Jimma Town Schools were contacted to acquire formal approval to undertake research activities at the selected schools. Each participant was needed to give written informed consent after the researcher explained the study’s nature, purpose, and methodology. Each participant signed the willingness confirmation after getting their approval. All study participants have been informed that the data will be treated as private and confidential and will only be used for research purposes. The participants were also made aware of their right to decline or leave at any time if they felt uncomfortable.

### Sampling procedure

According to Jimma Town Municipality, there are 13 urban kebele in Jimma Town. Besides, based on Jimma Town’s educational buero, there were 33 primary and 4 sary private schools, as well as 26 primary and 9 sary public schools. In these schools, there are 2689 female public primary (grades 7–8) and 542 sary students, as well as 792 female private elementary (grades 7–8) and 103 sary students. Four primary and two secondary public schools, as well as five primary and two secondary private schools, have been selected, a total of 13 schools were selected one from each kebele, using the lottery technique. The sample size for each selected school was set proportionally based on the number of female students in the school. Each participant female school student was picked at random from selected schools through a systematic random sampling procedure.

### Sample size determination

The single population proportion calculation was applied with an assumption to determine the number of female students to be included in the study. For Human Papillomavirus Vaccine Hesitancy Drives Low Coverage in Girls Attending Public Schools, the proportion was 67.1% in South Africa [19]. A single population proportion calculation based on the following assumptions: 95% confidence interval, 5% margin of error, and participant sample size were calculated as follows;

$$\begin{aligned} n = Z_{\frac{\alpha }{2}}^{2} \frac{P(1-P)}{d^{2}}, \end{aligned}$$

where

- The usual normal value $Z_{\frac{\alpha }{2}}$ = corresponds to the acceptable level of confidence.
- *d* denotes the precision error.
- *P* denotes an attribute’s estimated proportion.

$$\begin{aligned} n&= 1.96^{2} \frac{0.671(0.329)}{0.05^{2}}\\&= 339. \end{aligned}$$

After controlling for a 10% non-response rate, a sample size of 373 individuals was chosen for the study.

### Variables of the study

**Dependent variables:** Hesitancy of HPV

**Independent variables:** Socio-demographic variables such as Age, Religion, Student School Grade, parents’ Educational status, Parents’ occupation, Having older sister/older sister vaccinated, Participating in school mini-media club, Ownership of mobile phone, and School type (private/public). Awareness about HPV vaccine, Attitude towards the vaccine, Rumors, and vaccination history were considered. Besides, 5C Psychological Antecedents: Confidence, Complacency, Constraints, Calculation, and Collective responsibility

### Operational definitions

Human papilloma virus (HPV): A type of virus that can cause abnormal tissue growth (for example, warts) and other changes to cells. Infection for a long time with certain types of human papillomavirus can cause cervical cancer [20]. Human papilloma virus (HPV) Vaccine: a vaccination that aids in defending the body against contracting specific HPV types. Human papillomavirus vaccines are being used to prevent some of these cancers. They are also being used to prevent genital warts and abnormal lesions that may lead to some of these cancers. Also called the HPV vaccine [21]. Human papilloma virus (HPV) Vaccine Hesitancy: Delay in acceptance or refusal of HPV vaccines despite availability of vaccine services [22]. Attitude: Respondents’ attitudes toward HPV infection and vaccines were categorized as follows: negative if they had not answered correctly, and positive if they answered correctly. 5C Model: Five psychological antecedents of vaccination behavior represented in the 5C Model that measures vaccine hesitancy: confidence, complacency, constraints, calculation, and collective responsibility [23].

### Data collection procedure and techniques

A structured, pre-tested interviewer-administered questionnaire and semi-structured in-depth interview guide were prepared. The questionnaire was adapted from different studies done elsewhere [13]. To ensure consistency, the interview questionnaire for the female student was first translated into two native languages (Amharic and Afan Oromo) and back to English to check for its consistency. Data collectors were three college health students and there was one public health supervisor. The following techniques were used to collect data; For quantitative data to answer objectives one and two, which are to assess the level of hesitancy and associated factors related to HPV vaccine hesitancy, the questionnaire was divided into sections that were created to evaluate socio-demographic factors, parents socio-economic status, sources of information about HPV vaccination, vaccination history, opinion and attitude toward the HPV vaccine, and HPV vaccine hesitancy. The 5C psychological antecedents of vaccination data were collected using interview-administered questionnaires.

### Data quality assurance

To ensure data quality, the following operations were carried out: modifying questions from Standard tools and translating them into Amharic and Afaan Oromo. Data collectors were trained on how to use kobo tools, sampling procedures, interview tactics, and data collection processes, all while being overseen by a public health officer. The questionnaire was subjected to pre-testing to determine whether the questions were clear and suitable for extracting the necessary information and checking the understand-ability flow and consistency by taking 5% of samples from other schools which are not included in the actual data collection to evaluate the instrument’s ease of use. As a result, potential changes or modifications were evaluated at the time of data collection, and minor corrections on a few questions were included. The filled formats by the Kobo tool were checked.

### Data processing and analysis

The questionnaire’s consistency and completeness were examined. After data collection was made on Kobo Tool, download it in XLS format and export it to SPSS version 20 statistical software for cleaning and checking missing data. Analysis was made using Stata version 17 statistical software. After exporting the prepared data, descriptive statistics such as frequency distribution and measures of central tendency and variability were computed to describe the major variables of the study, while frequencies and proportions were computed for categorical variables. Bivariate analyses were used to analyze the initial crude relationship between each independent variable and dependent variable through the chi-square test. The significant independent variables with a P value of 0.25 were then transferred to multivariable analysis to control for confounders. The criteria for statistical significance were a P-value of 0.05, and AOR with a 95% confidence interval was used to illustrate the strength of the relationship.

## Results

### Socio-demographic characteristics

A total of 369 female school girls participated, with a 98.9% response rate. The respondents’ average age was 15.07 (14–17 years), and 249 (67.74%) of the participants were between the age of 14 and 15. In terms of religion, 126 (34.15%) were Muslim and 110 (29.81%) were Orthodox. Most 320 (86.72%) of students were living with their families. Besides, more than half of students used school mini-media and 245 (66.40%) of them own mobile phones. Of female participant students, 190 have an older sister, and 170 received the HPV vaccine, Table 1.

**Table 1:** Socio-demographic characteristics, family history and parent’s socio-economic status of female school students in Jimma town, Oromia, Ethiopia, 2023

Variables	Catagory	Frequency	Percent
Religion	Orthodox	110	29.81
	Muslim	126	34.15
	Protestant	107	29.00
	Others	26	7.05
Student grade level	7th	41	11.11
	8th	113	30.62
	9th	127	34.42
	10th	88	23.85
School type	Public school	256	69.37
	Private school	113	30.63
Guardian	Parents	320	86.72
	Relatives/others	49	13.28
Father educational level	Unable to read and write	130	35.23
	Able to read and write	38	10.30
	Primary grade 1–8	72	19.51
	Secondary grade 9–12	68	18.43
	Above grade 12	61	16.53
Mother educational level	Unable to read and write	125	33.88
	Able to read and write	27	7.32
	Primary grade 1–8	28	7.59
	Secondary grade 9–12	132	35.77
	Above grade 12	57	15.45
Guardian source of income	Merchant	246	66.67
	Government employee	76	20.60
	Others	47	12.74
Have an older sister	Yes	190	51.49
	No	179	48.51
Older sister vaccinated HPV vaccine	Yes	170	89.47
	No	20	10.53

### The overall HPV vaccine hesitancy status

**Fig. 1:**
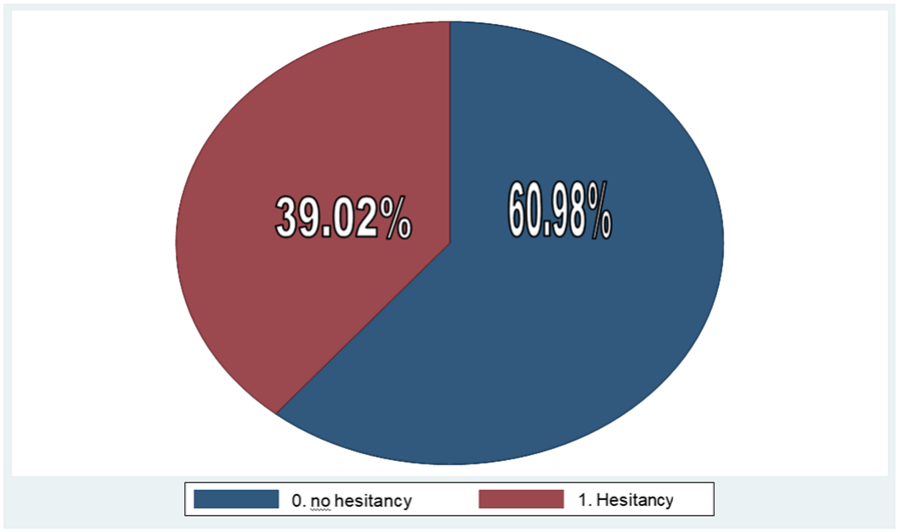
HPV vaccine hesistancy and no hesitancy group in Jimma town female school students, Ethiopia, 2023

For measuring the prevalence of hesitancy, ask the question, "If you get the chance of getting an HPV vaccine for free, do you want to receive the vaccination?" Two hundred twenty-five (60.98%) responded "yes" considered as "no hesitancy" or willing to take up the HPV vaccine without hesitancy and one hundred forty-four (39.02%) responded "no" considered "hesitancy" or refused for getting an HPV vaccination, Fig 1. According to this study, the prevalence of Hesitancy in HPV vaccine uptake in Jimma Town female school students was 39.02%. From 144 (39.02) hesitancy levels at the age of 11–13, at grade 6–7, individuals who were not participating in school- mini-media and had no smartphone accounted for greater than 50%. From Participants who stated an intention to receive HPV vaccination 225 (60.98%) the motives for going to be vaccinated were, Eighty-seven (38.66%) don’t want to be infected and Eighty-three (36.88%) believe in the efficacy of the HPV vaccine. Of those not yet intended to be vaccinated 144 (39.02%), reasons of refusal were, 64 (44.4%) Fearful of the vaccine’s mild side effects (e.g., fever, pain at the injection site) and major adverse effects (e.g., hospitalization, serious sickness), and 57 (39.58%) want to wait until they have more experience with these vaccines.

**Fig. 2:**
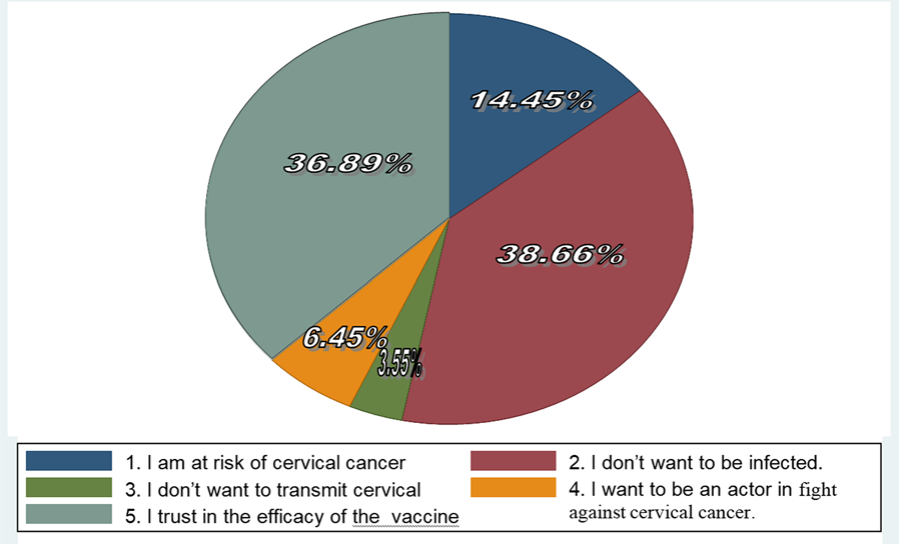
Reasons for receiving HPV vaccine (acceptance) in Jimma town female school students, Ethiopia, 2023

**Fig. 3:**
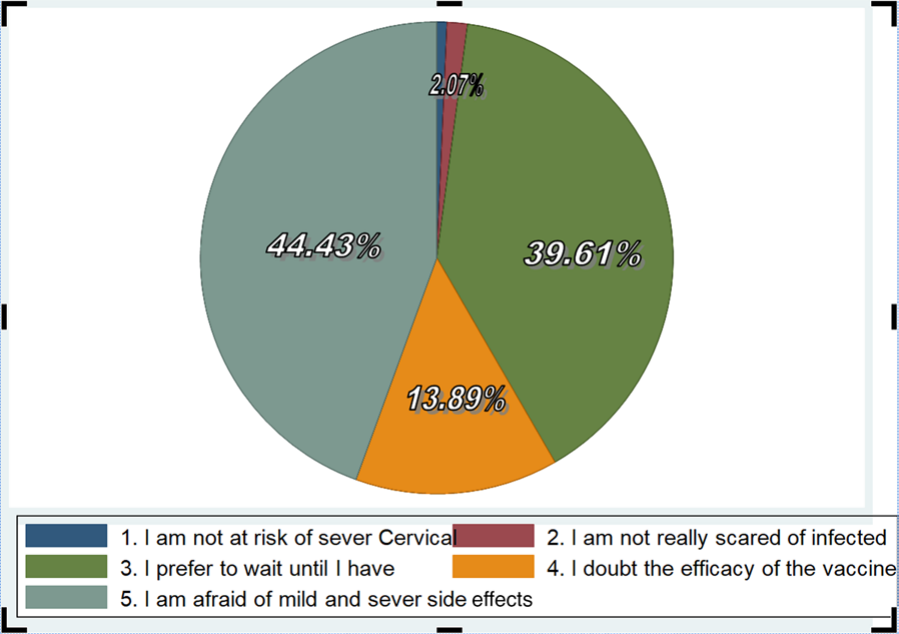
Reasons for not getting the HPV vaccine (acceptance) in Jimma town female school students, Ethiopia, 2023

Of 230 previously vaccinated female students, the most 174 (75.65%) have "no hesitancy" 104 students were vaccinated once time and 70 students were two times vaccinated, and the rest, only 56 (24.34%), have been vaccinated before with "hesitancy" status or were going to refuse if they get the chance of vaccination for the next time. If their family or friends supported their vaccination with the HPV vaccine, the majority two hundred fifty-five (69.10%) were surely or most probably going to vaccinate, and one hundred nine (29.53%) were surely or probably not going to vaccinate even though they were supported by family or friends. Besides, Reasons for receiving the HPV vaccine (acceptance) in Jimma town female school students, Fig. 2 and Reasons for not getting the HPV vaccine (hesitancy) in Jimma town female school students, Fig 3. Thus, figures very helpful for readers to understand the reason why they accept or refuse HPV vaccine.

## Awareness about HPV vaccine

Almost all female school students, 362 (98.10%), have reasonable information about the HPV vaccine, 131 (36.09%) and 103 (28.82%) heard about HPV from family or friends and schools, respectively. Regarding cervical cancer, 334 (90.51%) of them knew that HPV can cause cervical cancer, Table 2.

**Table 2:** Predictors associated hesitancy status with source of information about HPV vaccination among Jimma town schools, oromia, Ethiopia, 2023

Surce of Information	No hesitancy (N = 225)	Hesitancy (N = 144)	P-value
Heared of the HPV Vaccine			
Yes	220 (59.6%)	142 (38.48%)	0.567
No	5(1.35%)	2(0.54%)	
Where do you hear about HPV vaccine			
Social medias	53 (14.36%)	49 (13.27%)	0.002
Schools	77 (20.87%)	26 (7.04%)	
Religious groups	10 (2.71%)	2 (0.54%)	
Health care workers	1 (0.27%)	2 (0.54%)	
Family/friend	75 (20.32%)	57 (15.45%)	
Community member	9 (2.44%)	8 (2.16%)	
Heared of HPV cause cervical cancer			
Yes	202 (54.74%)	132 (35.77%)	0.546
No	23(6.23%)	12(3.25%)	

### Attitude towards HPV vaccination associated with hesitancy status

The majority of respondents, most of the 292 (79.13%) have positive attitudes towards the HPV vaccine, which successfully prevents cervical cancer. Of those, 181 (49.05%) were no hesitancy status group, and 264 (71.13%) have negative attitudes towards the HPV vaccine. This indicates that reasonable training should be given to school girls. The bivariate analysis indicates that it is significantly associated with hesitation status for the 153 (41.46) members of the no hesitancy status group. From participants as well as girls receiving the vaccine before their first sexual intercourse and receiving the HPV vaccine that is required for adolescents, 248 (67.20%) and 255 (69.10%) have favorable opinions, respectively. Both were significantly associated with hesitancy status and also not taking the vaccine because of lack of knowledge about it, 225 (60.97), and considering the HPV vaccine as unnecessary 233 (63.14), have Positive attitudes and made up their relationship to hesitancy status was strong, Table 3.

**Table 3:** Association between attitude towards HPV vaccination and HPV vaccination hesitancy status among Jimma town schools, Oromia, Ethiopia, 2023

The attitude to HPV vaccination	Category	Total N%	No hesitancy N%	Hesitancy N%	Chi-sq.	*p*-value
Cervical cancer prevented by HPV vaccine?	Yes	292 (79.13)	181 (49.05)	111 (30.08)	6.64	0.156
	No	77 (20.86)	44 (11.91)	33 (8.95)		
HPV vaccine educ should be provided to school	Yes	105 (28.45)	72 (19.51)	33 (8.94)	10.53	0.032
	No	264 (71.54)	153 (41.46)	111 (30.08)		
Should Girls vaccinated before first sexual intercourse?	Yes	248 (67.20)	146 (39.56)	102 (27.64)	16.44	0.002
	No	121 (32.79)	79 (21.40)	42 (11.38)		
Does Teenagers require information about HPV vaccine	Yes	255 (69.10)	152 (41.19)	103 (27.91)	17.47	0.002
	No	114 (30.89)	73 (19.78)	41 (11.11)		

Table 4 shows the distribution of 5Cs factors and their associations with vaccine hesitancy. Female students have low confidence in the safety and effectiveness of the vaccine, as shown by confidence-level questions. More than half (66, 17.88%), 102 (27.92%), and 214 (57.99%) disagreed with decisions made by public authorities that were in the best interests of the community. According to complacency questions, 202 respondents (54.01%) thought getting the HPV vaccine wasn’t necessary, and 123 respondents (33.33%) fell into the reluctance category. From the 252 individuals who agreed that regular learning stress prevents them from receiving vaccinations (68.78%), 113 (30.62) were in the group who expressed hesitation and their status was statistically significant.

**Table 4:** Chi-square results of 5Cs factors by hesitancy status among Jimma town schools, Oromia, Ethiopia, 2023

5C domain	Factors	Category	Total N%	No hesitancy N%	Hesitancy N%	Chi-sq.	p-value
Confidence	The safety and efficacy of the vaccine.	Distrust	102 (27.92)	36 (9.75)	66 (17.88)	19.18	0.001
		Neutral	23 (6.23)	13 (3.52)	10 (2.71)		
		Trust	244 (66.11)	176 (47.69)	68 (18.42)		
	Public authorities decide in the best interest of Community	Disagree	214 (57.98)	82 (22.13)	132 (35.76)	24.00	< 0.001
		Neutral	48 (13.00)	46 (12.46)	2 (0.54)		
		Trust	107 (28.99)	97 (26.28)	10 (2.71)		
Complacency	I think it is unnecessary to receive vaccinations as it cannot prevent HPV	Disagree	154 (41.72)	137 (37.12)	17(4.60)	21.26	< 0.001
		Neutral	13 (3.52)	9 (2.43)	4 (1.08)		
		Trust	202 (54.0)	79 (21.4)	123 (33.33)		
	I believe my immune system is powerful; it will protect me	Disagree	140 (37.93)	76 (20.59)	64(17.34)	18.54	0.001
		Neutral	19 (5.14)	14 (3.79)	1 (0.27)		
		Trust	210 (56.65)	135 (36.58)	64 (17.33)		
Calculation	Before to be vaccinated, against HPV, I weight the benefit and the risk to make best decision	Disagree	146 (39.56)	88 (23.84)	58 (15.71)	20.70	< 0.001
		Neutral	13 (3.52)	7 (1.89)	6 (1.62)		
		Trust	210 (39.83)	130 (22.22)	80 (17.61)		
	Before to be vaccinated, I prioritized whether its effective or not	Disagree	145 (39.29)	90 (24.38)	55 (14.9)	10.85	0.028
		Neutral	13 (3.52)	7 (1.89)	6 (1.62)		
		Trust	211 (57.17)	128 (34.68)	83 (22.48)		
Collective	I think vaccination against HPV is a collective action to prevent the spread of disease	Disagree	147 (39.83)	88 (23.84)	56 (14.46)	10.61	0.031
		Neutral	15 (4.06)	10 (2.71)	5 (1.35)		
		Trust	209 (56.63)	127(34.41)	82 (22.21)		
Constraints	Everyday learning stress may prevent me from getting vaccinated	Disagree	92 (24.93)	71 (19.24)	21 (5.69)	23.71	< 0.001
		Neutral	25 (6.77)	15 (4.06)	10 (2.71)		
		Trust	252 (68.78)	139 (37.66)	113 (30.62)		

### Factors associated with HPV vaccine hesitancy

Based on multivariable logistic regression analysis, Have smart phone, mother Higher educational level, older sister vaccinated, previously vaccinated of HPV vaccine, Confidence (Worried about safety and efficacy), complacency (unnecessary to receive HPV vaccination), constraints (learning stress not prevent from vaccination), and unbelieve in rumors (HPV vaccine ruins girls fertility) were found to be statistically significant predictors of HPV Vaccine Hesitancy at p-value < 0.05, Table 5.

**Table 5:** Factors associated with human papilloma virus vaccine hesitancy, in Jimma Town female school students, Oromia, Ethiopia, May, 2023

Factors	COR (95% CI)	p- value	AOR (95% CI)	p- value
Participate in school mini media (Rf: No)	...	...	...	...
Yes	0.44 (0.29, 0.68)	< 0.001	0.48 (0.35, 1.03)	0.061
Own Smart phone (Rf: No)	...	...	...	...
Yes	0.46 (0.29, 0.74)	0.001	0.51 (0.28, 0.94)	0.032
Older Sister vaccinated (Rf: No)	...	...	...	...
Yes	0.19(0.16,0.70)	0.012	0.04 (0.004, 0.42)	0.043
Previous vaccinated status (Rf: Not vaccinated)	...	...	...	...
Vaccinated at least one times	5.36 (3.39, 8.47)	< 0.001	0.64 (0.06 ,0.73)	< 0.001
Confidence (advice provided by medical staffs)(Rf: Trust)	...	...	...	...
Neutral	2.92 (1.63, 5.19)	< 0.001	1.17 (0.76, 1.87)	0.086
Distrust	2.94 (1.53, 5.64)	0.001	1.13 (1.12, 12.34)	0.066
Confidence (Trust in safty and efficacy) (Rf: Trust)	...	...	...	...
Neutral	0.89 (0.66, 8.75)	0.002	0.46 (0.87, 7.43)	0.235
Distrust	3.67 (1.60, 7.33)	0.001	3.21 (0.42, 13.45)	0.027
Complacency (vaccinations can’t prevent HPV)				
(Rf: necessary )	...	...	...	...
Neutral	0.94 (0.01, 0.97)	0.024	0.77 (0.025, 23.8)	0.885
Unnecessary	0.36 (0.18, 0.70)	0.003	2.37 (1.20, 4.67)	0.013
Complacency (cervical cancer not sever disease)				
(Rf: Not believe)	...	...	...	...
Neutral	0.17(0.08, 0.55)	0.011	0.11 (0.09, 1.21)	0.092
Believe	0.35 (0.18, 0.70)	0.003	0.43 (0.23, 1.32)	0.096
Before I get HPV vaccinated,				
I need to know more about this vaccine)				
(Rf: Disagree )	...	...	...	...
Neutral	0.58 (0.30, 1.12)	0.109	0.87 (0.43, 1.09)	0.201
Agree	0.18 (0.05, 0.66)	0.010	0.34 (0.09, 1.32)	0.054
Constraint (learning stress may prevent				
me from getting vaccinated) (Rf:prevent)	...	...	...	...
Neutral	0.52 (0.29, 0.94)	0.032	0.66 (0.34, 1.02)	0.062
Not Prevent	0.22 (0.10, 0.46)	< 0.001	0.36 (0.16, 0.81)	0.014
Belief in rumor (Vaccine ruins girls fertility) (Rf:Agree)	...	...	...	...
Disagree	0.66 (0.34, 1.06)	0.09	0 .21 (0.14,0.96)	0.036

## Qualitative study results

### Socio-demographic characteristics of the study participant

The qualitative study considered 13 parents of female students who are eligible for HPV vaccination. The majority of the study participants (76.9%) were female. Eight (61.53%) of the study participants were aged between 35 and 40 years, and the mean age was 31. Nine (69.2%) of the participants were housewives, and eight of them are unable to read or write (61.53%), Table 6.

**Table 6:** Socio-demographic characteristics of Parents/caregivers of school female students in Jimma town, Oromia, Ethiopia, 2023 (n = 13)

Variables	Category	Frequency	Percent
Age	25–30	1	7.69
	30–35	2	15.38
	35–40	10	61.53
Sex	Female	8	61.53
	Male	5	38.46
Educational status	Unable to read and write	8	61.53
	Able to read and write	3	23.07
	Primary and above	2	15.38
Occupational status	House wife	9	69.23
	Employer	4	30.76

### Theme I: safety and effectiveness

With the exception of one parent, practically every participant engaged in conversation regarding safety, according to the analysis of the data from the in-depth interviews. Nine (69.23%) of the parents we surveyed expressed concern regarding the vaccine’s efficacy and safety. Here are their comments: The first answer said, "I don’t have any faith in it; it is entirely harmful and useless, especially the vaccine given at school, which is unsafe. I don’t let my girls to get the vaccine because I’m worried about the long-term side effects and the vaccine’s expiration date, and I have no experience with or knowledge of it. The second respondent similarly voiced their opinion, saying," I don’t think the HPV vaccine is safe or effective at preventing HPV infection. Vaccinations might have unforeseen effects. I have not allowed my younger daughter to have vaccinations because my elder daughter’s reaction to the vaccine did not go away easily and she had pain at the time. In a similar vein, the other respondents feel as follows: "Regarding Cervical cancer prevention vaccination, it is unsafe and ineffective because cancer is uncommon in our society". Following is how the final respondent described their viewpoint: "I have gained some knowledge regarding the HPV vaccine from the community, but not enough from doctors and nurses. Because the responsible party didn’t send us a helpful message about it, I don’t believe it was secure. As she said, "I felt fear about the vaccine because of different rumors I have heard from the community, so I am badly felt to recommend it."

### Theme II: concern about side-effects

We asked the participants if they were worried about the negative consequences of the HPV vaccine. Under this theme, two categories were created: "I have positive concern about the side effects" and "I have a negative concern about the side effects". These issues were discussed below.

### Negative concern about the side effects (n = 8, 61.53%)

The majority of responders express concern about the adverse effects in their responses, which are summarized here. Of course, I have reservations, which is why I don’t want to immunize my daughters. Despite not knowing about the HPV vaccine at the time and having no personal experience with how the cervix and cancer are associated, I have not received the vaccine. Only childhood immunizations, in my opinion, are essential; I don’t believe they are helpful for anything else. The other respondents also stated, “I have negative concerns about the vaccines because they are all delivered from developed countries to us, yet in Western countries, the vaccine is given with paid money, so why do they offer them to us freely? In general, I believe the shots were outdated or expired”. Additionally, a Reasonable number of responders are really worried about the adverse effects. I am worried about the vaccine’s side effects because people in my neighborhood have told me it can paralyze the hand where it was injected, Table 6.

### Positive concerned about the side effects (n = 5, 38.46%)

The majority of those surveyed believe the following. "Even though my daughter has not yet received her vaccinations, I strongly advise that she do so at the appropriate time as I believe that all girls should, even though every vaccine and medication has side effects". Respondents from the father’s respondents concur. “In my perspective, all vaccines have no significant negative effects because the government forbade their administration to all females if there were any potential health risks. The health minister is doing a terrific job of making the HPV vaccine publicly available, naturally with little adverse effects”.

### Theme III: awareness or information

The participants were questioned regarding the HPV vaccine’s informational sources and the degree of knowledge held by those who had heard of it. The vast majority of individuals acknowledged that they don’t know enough about the vaccine. The specifics are listed below.

### Not have/get information (n = 8, 61.53%)

Insufficient knowledge about the HPV vaccine is reported by 61.53 % of responders. The thoughts of five responders are similar. "I haven’t had any experience with this vaccine, but I have heard about it on TV and in the neighborhood, of course. I don’t believe my daughter will likewise receive vaccinations because I don’t have adequate information from medical professionals or other authoritative entities. The other three responders concurred with this viewpoint. "I didn’t know there was a vaccination against cervical cancer until last week when they came to their schools to vaccinate, and also because my daughter hasn’t received enough information about it; they simply say it is a vaccine against cervical cancer without developing enough awareness to girls in each class, so I have no awareness about it at all," my daughter said.

### Have/get information (n = 5, 38.46%)

A total of 38.46% of the respondents verified having sufficient knowledge about the HPV vaccine. Here is their assessment on it. "I read about the vaccine on social media and learned about it from television. It protects girls aged 9 to 14 from cervical cancer and other cancers of a similar nature. I believe I’ve learned enough about it from the media". The male reply, a father, also verified the information’s source: "I think I know something about this vaccine. I learned that it is against cervical cancer for girls through various media, the local community, and friends. This needs to be provided even before the start of sexual contact. It is helpful in my opinion in preventing HPV and associated disorders".

### Theme IV: factor influencing HPV vaccine hesitancy

The participants were questioned regarding the causes of HPV vaccine hesitancy as well as the reasons why some parents choose to delay or forego immunizing their daughters. The causes of their daughters’ reluctance to receive the HPV vaccine have been clearly identified by their parents. The following are the potential risk reasons that present a challenge to not receiving the vaccine, in the parents’ beliefs and opinions: lack of knowledge and information about the vaccine, questions about long-term safety, religious considerations, false information about the vaccine, and worries about the adverse effects. As a result, going to female students who are not supported by their families is one of the variables linked to HPV vaccine hesitation. The majority of respondents were not aware their daughter was at danger of any HPV-related condition, such as cervical cancer, if she had not received the vaccine. The following sampling responses provided more support this finding. "In my opinion, the majority of parents lack knowledge and understanding about the vaccine, religious concerns, and the cultural tendency to keep the immunization program the same other than for childhood vaccination may reduce vaccine uptake and increase skepticism". The other respondents agree, stating the following: "I think this is due to a lack of adequate and satisfactory information from health professionals or other responsible bodies; other reasons include various rumors about vaccinations heard in the community, which are not denied by any health professionals or other bodies; and lastly, people do not trust the government’s vaccination program, particularly those administered in schools".

According to authors, parents delay or refuse to immunize their daughters for these and other reasons. The effects of COVID-19 have been attested by three respondents. "In my view, there are additional reasons for HPV vaccine reluctance in addition to ignorance: the COVID-19 pandemic outbreak and the overwhelming focus on COVID-19 prevalence as opposed to other diseases or vaccine-related issues."

## Discussion

Despite the fact that the WHO has listed vaccine hesitancy as one of the top ten risks to health, particularly in low-income countries, there have been no realistic prior studies in Ethiopia or the research area. In fact, studies being carried out in other regions of the world, but very limited literature were published in Ethiopia specifically in the study area. Due to this fact, this study employed a mixed-methods approach, researchers were able to theoretically and analytically combine unique findings from qualitative research (from in-depth interviews of parents) and easily replicable from cross-sectional interviews with female students.

Thus, the present paper has focused on assessing the prevalence of Human Papilloma Virus Vaccine Hesitancy and associated factors. In this study prevalence of hesitancy in HPV vaccine uptake is 39.02%. From those who did not intend to be vaccinated their reasons for refusal were afraid of mild side effects (e.g., fever, pain at the injection site) and serious side effects (e.g., hospitalization, serious illness) of the vaccine, this is consistent with research done in the central Ethiopian city of Ambo [24]. This study found that concerns about side effects were the most common vaccine barriers. The discomfort of getting vaccinated was another concern[24]. Similarly, 57 people (39.58%) would rather hold off until they know more about these vaccinations. According to research done at a university in China, there are a lot of people who are reluctant to get the HPV vaccine, with the rate being 10.3% [25].

The amount of hesitation shown in the present study, however, is lower when compared to a study done in South Africa on females enrolled in public schools (69.6%) [19]. This mismatch may be caused by variances in sociodemographic characteristics, community knowledge, or literacy levels. A study conducted in Colombia with parents and schoolchildren found that 57.1% of participants had not started immunization, which is higher than this study [26]. This difference may be due to the study’s smaller sample size and different sociodemographic profiles. Youths in Switzerland participated in another research effort that revealed a prevalence of HPV vaccine reluctance of 31% [27], which is somewhat compatible with the results of the current study.

Additionally, the aforementioned variance for trials conducted outside of Ethiopia may reflect a genuine difference in the vaccine’s accessibility and availability. In particular, in public schools, the health professions simply vaccinate those who are willing and do not take any measures to influence the hesitant students. As a result, those students do not trust the vaccination and the health professions. This is evidenced during the HPV vaccination period when there is no awareness creation or giving enough information about the vaccine to increase the up taking level. According to the study’s qualitative findings, reluctance about the HPV vaccine was substantially correlated with confidence, which includes trust in the health care providers.

Concerns over the HPV vaccine among parents are very severe. "Even about the vaccination, I heard from my daughter last week when they came to their schools to vaccinate, but I didn’t hear about it before and I didn’t know there is a vaccination against cervical cancer, and also, my daughter hasn’t gotten any information about it; they simply say it is a vaccine against cervical cancer but nothing awareness to girls in each class, so I have no awareness about it at all". The goal of this study was to identify the most significant causes of HPV vaccine hesitancy reported in the literature. Although a cause-and-effect link could be demonstrated, similar findings from other studies were reported.

Significant factors that influence the uptake of the HPV vaccine were found in this investigation. These include the following: smartphones, mothers with higher educational levels, older sisters who have received vaccinations, having received vaccinations at least once before, confidence (not believing in the safety and efficacy of the vaccine), not believing in rumors that the HPV vaccine harms girls’ fertility, complacency (HPV vaccination is not necessary), and restrictions (learning stress prevents from receiving the vaccine). These studies provide important advice for the hesitation of the HPV vaccine.

Vaccine against HPV Girls who own smartphones reduce vaccine reluctance by 51% compared to those who don’t, suggesting that smartphone ownership is highly connected with hesitancy. This showed that using a smartphone is a protective factor for HPV vaccination reluctance, which is connected with it or a 49 % reduced risk for HPV vaccine, and this finding was corroborated by [28]. According to a variety of academic studies, mobile devices can make HPV vaccination decision-making aids broadly accessible on digital platforms, reducing vaccine reluctance. This is supported by the findings of a completed telephone survey study. In contrast to parents of girls without phones, who are probably more hesitant, this study indicated that parents of girls with mobile phones are more likely to get the HPV vaccine and are better aware about it [29]. A correlation between vaccine reluctance and social media knowledge about vaccinations was also suggested by other scientific theories.

Mother’s educational level and the HPV vaccine hesitancy were substantially correlated. In this study, women with higher educational status (those who have completed secondary school and above) decrease vaccine reluctance by reducing the risk of HPV vaccine uptake by 53% or 47% compared to mothers with lower educational status [30]. Children under the age of 16 and school girls whose parents had lower education levels demonstrated considerably higher vaccine hesitation, per this study [30]. People with at least a higher education level have more opportunity to have adequate information of HPV and its vaccines, which leads to less reluctance to get the HPV vaccine. Additionally, a high level of education can considerably improve understanding, enhance the likelihood that someone will receive appropriate information, and have a protective influence on vaccine reluctance. According to science, moms with high levels of education and literacy tend to have girls who are less reluctant to get immunized. This is due to the fact that mothers who are knowledgeable about vaccinations are more inclined to insist that their kids get them [31].

### Strength and limitation

The strength of the present paper is, mixed quantitative and Qualitative approaches were considered to answer "why questions and also, and both school girl and their parents participated. The limitation of this study was considered only one deliberately chosen town and hence, it is not shown the national picture".

## Conclusion

The current study was able to pinpoint various factors that contributed to vaccine reluctance for the HPV virus. Although vaccine resistance is a complicated issue, there isn’t any solid proof that any particular strategies are working to address it. Both parents and schoolgirls’ attitudes regarding getting the HPV vaccine and their levels of awareness are relatively low. They have formed false impressions regarding the side effects and associated impacts. Health professionals should provide the necessary training to parents and schoolgirls in order to enhance and maximize their knowledge, attitudes, and awareness levels. Delivering brief and precise reading leaflets, brochures, and text messages via smartphone may be realistic to inform and update both parents and schoolgirls. Timely and basic information should be provided by mainstream mass media, and school mini-media.

## Data Availability

The data sets analyzed in this study available from the corresponding author on reasonable request. The R code used to analyze the data provided as a supplement of the article.

## Abbreviations

AOR: Adjusted odds ratio
COR: Crude odds ratio
HPV: Human papillomavirus
STD: Sexually transmitted disease
WHO: World health organization
AF: Attributable fraction
FDA: Food and Drug Administration
GAVI: Global alliance for vaccines
WLWH: Women live with human immunodeficiency virus

## References

[1] Markowitz LE, Schiller JT (2021). Human papillomavirus vaccines. J Infect Dis.

[2] Owh. Human papillomavirus (HPV) [Internet]. Available from: www.twitter.com/WomensHealth.

[3] Oshman LD, Davis AM (2020). Human papillomavirus vaccination for adults: updated recommendations of the advisory committee on immunization practices (ACIP). JAMA J Am Med Assoc.

[4] Bogani G, Lalli L, Sopracordevole F, Ciavattini A, Ghelardi A, Simoncini T, Plotti F, Casarin J, Serati M, Pinelli C (2022). Development of a nomogram predicting the risk of persistence/recurrence of cervical dysplasia. Vaccines.

[5] Chen L, Huang L, Dong B, Gu Y, Cang W, Li C, Sun P, Xiang Y (2023). ADCY7 mRNA is a novel biomarker in hpv infection and cervical high-grade squamous lesions or higher. Biomedicines.

[6] Tucci Chiara Di, Schiavi Michele Carlo, Faiano Pierangelo, D’Oria Ottavia, Prata Giovanni, Sciuga Valentina, Giannini Andrea, Palaia Innocenza, Muzii Ludovico, Panici Pierluigi Benedetti (2018). Therapeutic vaccines and immune checkpoints inhibition options for gynecological cancers. Crit Rev Oncol Hematol..

[7] de Martel C, Plummer M, Vignat J, Franceschi S (2017). Worldwide burden of cancer attributable to HPV by site, country and HPV type. Int J Cancer..

[8] ICO. Human Papillomavirus and Related Diseases Report. 2016;(October). Available from: www.hpvcentre.com

[9] ICO/IARC Information Centre on HPV and Cancer. Ethiopia Human Papillomavirus and Related Cancers, Fact Sheet 2023 (2023-03-10).

[10] Bruni L, Diaz M, Castellsague X, Ferrer E, Xavier Bosch F, De Sanjose S (2010). Cervical human papillomavirus prevalence in 5 continents: meta-analysis of 1 million women with normal cytological findings. J Infect Dis..

[11] Mahlet. II. Complementary data on cervical cancer prevention Figure 2. Estimated coverage of cervical cancer screening in Ethiopia, by age and study [Internet]. 2021. Available from: www.hpvcentre.net

[12] Ukumo EY, Woldehawariat FG, Dessalegn SA, Minamo DM, Ukke GG (2022). Assessment of knowledge about human papillomavirus vaccination among primary school girls in Arba Minch Town, South Ethiopia, 2020 an institution-based cross-sectional study. Cancer Manag Res..

[13] Lakneh EA, Mersha EA, Asresie MB, Belay HG (2022). Knowledge, attitude, and uptake of human papilloma virus vaccine and associated factors among female preparatory school students in Bahir Dar City, Amhara Region, Ethiopia. PLoS One.

[14] Biyazin T, Yilma A, Yetwale A, Fenta B, Dagnaw Y (2022). Knowledge and attitude about human papillomavirus vaccine among female high school students at Jimma town, Ethiopia. Hum Vacc Immunother.

[15] Biyazin T, Yetwale A, Fenta B (2022). Willingness to accept human papillomavirus vaccination in Jimma town, Ethiopia. Hum Vacci Immunother.

[16] SAGE Working Group on Vaccine Hesitancy. http://www.who.int/immunization/sage/sagewg vaccine hesitancy apr12/en/. Accessed 02 Feb 15.

[17] Farhart CE, Douglas-Durham E, Lunz Trujillo K, Vitriol JA. Vax attacks: how conspiracy theory belief undermines vaccine support. In: Progress in molecular biology and translational science. Elsevier Inc.; 2022; 135–169 p. 1st edn. vol. 188. 10.1016/bs.pmbts.2021.11.001. 10.1016/bs.pmbts.2021.11.001 PMC8713072 35168741

[18] MacDonald NE, Eskola J, Liang X, Chaudhuri M, Dube E, Gellin B (2015). Vaccine hesitancy: definition, scope and determinants. Vaccine..

[19] Khosa LA, Meyer JC, Motshwane FMM, Dochez C, Burnett RJ (2022). Vaccine hesitancy drives low human papillomavirus vaccination coverage in girls attending public schools in South Africa. Front Public Health..

[20] Topics H. Human papillomavirus (HPV). 2006.

[21] WHO. Guide to introducing HPV vaccine into National Immunization Programmes. World Health Organ. 2016;91.

[22] Chowdhury S, Pratim CP. Universal health coverage—there is more to it than meets the eye. J Fam Med Prim Care. 2017;6(2),169–70. http://www.jfmpc.com/article.asp?issn=2249-4863;year=2017;volume=6;issue=1;spage=169;epage=170;aulast=Faizi 10.4103/jfmpc.jfmpc_13_17 PMC5629889 29026777

[23] Adeyanju GC, Sprengholz P, Betsch C, Essoh TA (2021). Caregivers-willingness to vaccinate their children against childhood diseases and human papillomavirus: a cross-sectional study on vaccine hesitancy in Malawi. Vaccines..

[24] Beyen MW, Bulto GA, Chaka EE, Debelo BT, Roga EY, Wakgari N (2022). Human papillomavirus vaccination uptake and its associated factors among adolescent school girls in Ambo town, Oromia region, Ethiopia, 2020. PLoS One.

[25] Huang Y, Chen C, Wang L, Wu H, Chen T, Zhang L (2022). HPV vaccine hesitancy and influencing factors among University Students in China: a cross-sectional survey based on the 3Cs Model. Int J Environ Res Public Health..

[26] Cordoba-sanchez V, Lemos M, Tamayo-lopera DA, Gorin SS. HPV-vaccine hesitancy in Colombia : a mixed-methods study. 2022;1-16. 10.3390/vaccines10081187 PMC9332743 35893836

[27] Kiener LM, Schwendener CL, Jafflin K, Meier A, Reber N, Schärli Maurer S (2022). Vaccine hesitancy and HPV vaccine uptake among male and female youth in Switzerland: a cross-sectional study. BMJ Open..

[28] Woodall WG, Zimet G, Kong A, Buller D, Reither J, Chilton L (2021). Vacteens.org: a Mobile Web app to improve HPV vaccine uptake. Front Digit Health..

[29] Gauna F, Verger P, Fressard L, Jardin M, Ward JK, Peretti-Watel P (2023). Vaccine hesitancy about the HPV vaccine among French young women and their parents: a telephone survey. BMC Public Health.

[30] Yu Y, Xu M, Sun J, Li R, Li M, Wang J (2016). Human papillomavirus infection and vaccination: awareness and knowledge of HPV and acceptability of HPV vaccine among mothers of teenage daughters in Weihai, Shandong, China. PLoS One..

[31] Mihretie NG, Tewachew ML, Alemu DA, Habtamu GB, Tigist SY, Agernesh DM (2022). Knowledge and willingness of parents towards child girl HPV vaccination in Debre Tabor Town, Ethiopia: a community based cross-sectional study. Reprod Health.

